# Supplementary material for: Content-rich biological network constructed by mining PubMed abstracts
Source: BMC Bioinformatics. 2004 Oct 8;5:147. doi: 10.1186/1471-2105-5-147 (PMC528731; doi:10.1186/1471-2105-5-147)
Supplement: Additional File 2 — The original results of the above study (non-essential files are deleted to keep the file size under the limit set by BMC bioinformatics). [file 1471-2105-5-147-S2.bz2 › chilibotAdditionalFile2/dip20/452ID10477718E10463/html/CCR5_CCL13.html]

 


 **CCR5** and **CCL13** 
  
Found 14 abstracts in PubMed, retrieved 14.  
 

 What does Google say? 
 PDF only 
| .edu only 

---

**Interactive relationship** (e.g. stimulation, inhibition, etc)

**Neutral relationship**- MCP 4  [ **CCL13** ]  also bound to  **CCR5**  with high affinity and was efficiently displaced by other  **CCR5**  ligands.  Ref: 9790730 Cell Immunol, 1998
- CCR3,  **CCR5**  and CCR2 expressing leukocyte populations were identified at sites of MCP 4  [ **CCL13** ]  expression.  Ref: 11400154 J Pathol, 2001
- **CCR5**  is a functional receptor for MIP 1alpha, MIP 1beta, RANTES regulated on activation normal T cell expressed, MCP 2, and MCP 4  [ **CCL13** ]  and constitutes the main coreceptor for macrophage tropic human and simian immunodeficiency viruses.  Ref: 10574939 J Biol Chem, 1999
- **CCR5**  is most closely related to CCR2b, another chemokine receptor that responds to monocyte chemoattractant protein MCP 1, MCP 2, MCP 3, and MCP 4  [ **CCL13** ] .  Ref: 9312096 J Biol Chem, 1997
- MIP 1alpha, MIP 1beta, RANTES, MCP 2 and MCP 4  [ **CCL13** ]  were able to stimulate increases in intracellular calcium ions via activation of  **CCR5**  whereas MCP 3 was without effect.  Ref: 11861332 Br J Pharmacol, 2002

**Non-interactive relationship** (e.g. studied together, co-existance, homology, etc.)

- Recently we reported that the chemokines MIP 1alpha, MIP 1beta, and RANTES secreted by BM derived CD34 cells may compete with the macrophagotropic HIV R5 HIV strain for the  **CCR5**  coreceptor.In this study we extended our previous observations and examined various lympho hematopoietic CD34 cells isolated from thymus Th, cord blood CB, mobilized peripheral blood mPB, and BM for the expression of beta chemokines binding to  **CCR5** , i.e., MIP 1alpha, MIP 1beta, RANTES, MCP 2, MCP 3, and MCP 4  [ **CCL13** ] , and the alpha chemokine SDF 1 binding to CXCR4 as these chemokines may compete with the R5 and X4 HIV strains, respectively, for entry into cells.We found that Th, CB, mPB, and BM derived CD34 cells express mRNA transcripts for all the beta chemokines tested but not for SDF 1.  Ref: 11146155 Exp Hematol, 2000
- MCP 4  [ **CCL13** ]  induced a calcium flux in HEK 293 cells transfected with the monocyte selective MCP 1 receptor CCR 2B and the eosinophil selective eotaxin receptor CCR 3, but not in the more widely expressed CCR 1 or CCR 5  [ **CCR5** ] .  Ref: 8955214 J Immunol, 1996
- In order to evaluate if these cells are infectabile by human immunodeficiency virus HIV and could be employed as a model in HIV research we exposed these cell lines to X4 T cell tropic and R5 macrophage tropic and subsequently tried to correlate their infectability with i level of chemokine coreceptor CXCR4 and  **CCR5**  expression, ii coreceptor functionality calcium flux, chemotaxis and phosphorylation of MAPK p42 44 and AKT and iii endogenous expression and secretion of HIV related chemokines which compete with the virus for binding to CXCR4 SDF 1 CXCL12 or  **CCR5**  MIP 1beta CCL4, MIP 1alpha CCL3, RANTES CCL5, MCP 2 CCL8, MCP 3 CCL7 and MCP 4  [ **CCL13** ]   **CCL13** .  Ref: 11737246 Eur J Haematol, 2001
- We investigated the effects of a number of naturally occurring chemokines MIP 1alpha, MIP 1beta, RANTES, MCP 2, MCP 3, MCP 4  [ **CCL13** ]  on different processes linked to the chemokine receptor  **CCR5**  in recombinant CHO cells expressing the receptor at different levels.  Ref: 11861332 Br J Pharmacol, 2002
